# Pain Levels after Local Anaesthetic with or without Hyaluronidase in Carpal Tunnel Release: A Randomised Controlled Trial

**DOI:** 10.1155/2015/784329

**Published:** 2015-10-26

**Authors:** G. Yeo, A. Gupta, G. Ding, H. Skerman, M. Khatun, D. Melsom

**Affiliations:** ^1^Royal Brisbane Hospital, Australia; ^2^Ipswich Hospital, Australia; ^3^University of Queensland, Australia

## Abstract

*Purpose*. Hyaluronidase is an enzyme that temporarily liquefies the interstitial barrier, allowing easy dispersal of local anaesthetic through cleavage of tissue planes. This prospective, blinded, randomised controlled study investigates the utility of adding hyaluronidase to local anaesthetic in the setting of carpal tunnel release. *Methods*. 70 consecutive carpal tunnel release patients were recruited and randomised into a control group only receiving local anaesthetic and a hyaluronidase group receiving both hyaluronidase and local anaesthetic. Pain scores were rated using the visual analogue scale (VAS) by patients immediately after local anaesthetic injection and again immediately after the carpal tunnel release. *Results*. Preoperative VAS scores, taken after local anaesthetic injection, were greater than postoperative VAS scores. Postoperative VAS scores were significantly lower in the hyaluronidase group and tourniquet times were significantly shorter in the hyaluronidase group. *Conclusion*. Hyaluronidase addition to local anaesthetic in carpal tunnel release resulted in significant reductions in operative time and pain immediately after operation.

## 1. Introduction

Carpal tunnel release is routinely performed under local anaesthetic (LA) and patients find local anaesthetic injection the most painful part of the procedure [1, 2]. The fibrous anatomy of the carpal region makes tissue tension high when local anaesthetic is injected, and this tension is thought to contribute to injection pain [3, 4]. Since hyaluronidase aids in the dispersal of local anaesthetic through tissue planes, we propose that it may reduce injection pain through the subcutaneous tissues, as measured with the visual analogue scale (VAS).

Hyaluronan and chondroitin, both depolymerised by hyaluronidase, are the major components of the intercellular cement, or “ground substance,” which binds collagen, connective tissue, and proteins together. Hyaluronidase reversibly liquefies this interstitial barrier [5–7] and is routinely used in ophthalmic surgery where it disperses the local anaesthetic and reduces the pressure behind the globe [8, 9]. It also reduces the volume of local anaesthetic required by 2.4-fold [10] and protects against local anaesthetic myotoxicity [11, 12]. Hyaluronidase has also been shown to have antiscar and antiadhesion properties which may be of theoretical benefit in the carpal tunnel release setting [13].

Many factors influence the infiltration pain of local anaesthetic, and simple measures such as minimising volume, slow infiltration, and long, small diameter needles can be helpful. The acidity of local anaesthetic, cold temperature, and tissue tension have been shown to be important [3, 4, 14–16]. In particular, in carpal tunnel release, Watts and McEachan, 2005, successfully reduced injection pain through the use of dental needles; however, there have been mixed results in trials of alkalisation and warming in this region [17, 18]. To the authors' knowledge, there have not been many studies examining the effect of hyaluronidase on injection pain in carpal tunnel release, although hyaluronidase use in this setting is described [19].

## 2. Patients and Methods

Initial protocol design and power analysis was followed by obtaining ethical approval from the West Moreton Health Service District Human Research Ethics Committee. The design of this study was in accordance with the 2010 CONSORT guidelines [20].

Initially, seventy consecutive patients were recruited and informed consent was obtained at a single orthopaedic tertiary referral hospital. Eligibility criteria included all patients with carpal tunnel syndrome confirmed either clinically or through nerve conduction studies over a 1 year period from February 2011 to February 2012. Subsequently, patients were randomised to hyaluronidase or control groups by a random number generator. Randomisation was concealed via envelopes.

No patients were excluded before randomisation; however, 9 patients were excluded after randomisation; 4 patients were in the hyaluronidase group and 5 patients were in the control group. Six patients were excluded due to insufficient data collection, 1 patient required conversion to a general anaesthetic, 1 patient complained of severe tourniquet pain, and 1 patient required anaesthetic sedation during injection making their VAS score's unreliable. This left us with a total number of 61 patients for the study (Table 1).

All investigators in the trial were blinded until the data collection was completed. The anaesthetist was responsible for mixing the local anaesthetic, with or without added hyaluronidase powder. As hyaluronidase powder dissolves as a clear solution into the local anaesthetic, there was no discernible difference between the syringe contents; neither was there any visible difference in tissue appearance after injection, thus allowing the investigators to remain blinded.

The hyaluronidase trial group injection contained 3.5 mL of the longer-acting levobupivacaine (Chirocaine) 75 mg/mL, 3.5 mL of the shorter-acting Xylocaine 1%, and 1500 international units of hyaluronidase powder (Hyalase, Sanofi Aventis, Australia). The control group injection contained the same volume and concentration of local anaesthetic without hyaluronidase. The full volume of the injections was given via a single puncture site at a slow rate with a 25-gauge needle, specifically into the subcutaneous tissues along the length of the proposed incision, all performed by a single surgeon (Level III), who then assisted different surgeons (Level I) in performing the carpal tunnel release [21].

A VAS pain score documenting the level of pain after injection was obtained immediately after LA infiltration (pre-op VAS scores) and at the end of the carpal tunnel release (post-op VAS scores). A tourniquet was used in all operations and the length of tourniquet times was recorded from inflation to deflation.

Statistical analysis was performed using the Wilcoxon signed-rank test, Mann-Whitney *U* test, Spearman's correlation test, and independent sample *t*-test as appropriate.

## 3. Results

Postoperative VAS scores (taken at the end of the procedure) were overall amongst both groups combined significantly compared with the pre-op (immediately after injection) VAS scores (*p* value = 0.032). The difference in pain scores in the hyaluronidase group was not statistically significant immediately after injection (pre-op VAS; median score 3.00 versus 3.98; *p* value = 0.250). However, the reduction in pain score postoperatively was statistically significant (post-op VAS; median score 1.45 versus 2.87; *p* value 0.030). This represents a mean reduction in postoperative VAS score of 1.42. The change in VAS score was obtained comparing the VAS score at the end of the injection of local anaesthetic and at the end of the carpal tunnel release. Patients from the control group experienced on an average a smaller change in VAS score (median score 0.38) compared to the treatment group (median score 1.20), though this difference was not statistically significant (*p* value = 0.532) (Table 2).

The hyaluronidase group experienced a statistically significant shorter tourniquet time of 10.2 (±2.3 minutes) compared to the control group with tourniquet time of 11.8 ± 2.9 minutes. This equates to the hyaluronidase group experiencing a mean difference of 1.5 minutes shorter tourniquet time with a *p* value of 0.027 (Table 3). Tourniquet time, whether reflective of tourniquet pain or simply a longer operation, appeared to be contributory to the overall patient experience of pain. The correlation between tourniquet time and post-op VAS was not significant when each of the control and hyaluronidase groups were examined separately (Table 4).

## 4. Discussion

This study demonstrates the potential promise of adding hyaluronidase in carpal tunnel release with small but significant reductions in postoperative pain and tourniquet time. Postprocedure pain was less than infiltration pain (*p* value = 0.032), confirming that injection pain was the most painful part of the procedure. Similar results have been seen in 56 carpal tunnel releases performed by Lawrence and Desai, 2002. These authors also used tourniquets for all surgeries. While they found that VAS scores after surgery were less than those at needle insertion or anaesthetic injection, they did not comment on the significance of this finding.

There was a statistically significant reduction in the post-op VAS scores between the hyaluronidase and control groups; this was possibly justified by the cleavage of tissue planes from hyaluronidase allowing greater infiltration of the local anaesthetic over a greater area providing greater relief of pain. The addition of hyaluronidase can also significantly enhance the area of anaesthesia achieved by local anaesthetic infiltration, decreasing the amount of tissue distortion without decreasing its efficacy. This has been noted in previous papers which observed increased surface area of local anaesthesia with the addition of hyaluronidase [22, 23].

It is promising to note that there was a significant difference in shorter operation (i.e., tourniquet) times in the hyaluronidase group (10.2 ± 2.3 minutes) compared to the control group (11.8 ± 2.9) with a *p* value = 0.027. Though there are multiple reasons for this, a possible explanation may be again the ease of dissection due to clarification of the tissue planes, one of the benefits in dermatologic surgery emphasized by Clark and Mellette Jr. [24].

This paper demonstrates that with a significant reduction in both post-op VAS scores and in shorter operating times, hyaluronidase would appear to be an effective additive to local anaesthetic infiltration during carpal tunnel releases. Addition of hyaluronidase has the potential to allow for a greater surface area of anaesthesia [22, 23] potentially due to its cleavage of tissue planes, allowing ease of surgical dissection during carpal tunnel release explaining its shorter operating time.

Future considerations for this paper would include a cost-benefit analysis compared to local anaesthesia alone plus its potential benefits in wound healing.

## Figures and Tables

**Table 1:** Total sample numbers before and after randomization including exclusions.

	Total sample	Control	Hyaluronidase
Before randomization	70	35	35
After randomization	61	30	31
Exclusions	9	5	4

**Table 2:** Pre- and post-op VAS scores including VAS change scores in control versus hyaluronidase.

Group	Sample	Median (IQ)	*p* value
*Overall *			
Pre-op VAS	61	3.68 (1.99, 4.70)	0.032
Post-op VAS	61	2.00 (0.50, 4.85)	
*Pre-op VAS*			
Control	30	3.98 (2.00, 5.05)	0.250
Hyaluronidase	31	3.00 (1.05, 4.70)	
*Post-op VAS*			
Control	30	2.87 (0.89, 5.29)	0.030
Hyaluronidase	31	1.45 (0.04, 3.01)	
*VAS change*			
Control	30	−0.38 (−2.74, 2.00)	0.523
Hyaluronidase	31	−1.20 (−3.00, 0.84)	

**Table 3:** Tourniquet time in control versus hyaluronidase.

Tourniquet time	Sample	Mean tourniquet time in minutes (SD)	Mean difference (95% CI)	*p* value
Control	30	11.8 (2.9)	1.5 (0.18, 2.9)	0.027
Hyaluronidase	31	10.2 (2.3)		

**Table 4:** Correlation coefficient between tourniquet time and post-op VAS scores between control and hyaluronidase.

Group	Sample	Correlation coefficient (tourniquet time versus post-op VAS)	*p* value
Overall	59	0.23	0.084
Control	30	0.09	0.635
Hyaluronidase	29	0.31	0.101
